# Attitude and knowledge about electroconvulsive therapy (ECT) among medical students in Nepal: a pilot survey

**DOI:** 10.1192/bjo.2021.444

**Published:** 2021-06-18

**Authors:** Suresh Thapaliya, Shizu Singh, Shuva Shrestha, Anoop Krishna Gupta

**Affiliations:** 1 Kent and Medway NHS and Social Care Partnership Trust; 2 National Medical College and Teaching Hospital

## Abstract

**Aims:**

Electroconvulsive Therapy (ECT) is an important modality of treatment for treatment resistant psychiatric disorders. Young medical students like general public might harbor several misconceptions about ECT. In this pilot survey, we explored the knowledge and attitude about ECT amongst young medical students training in a medical college affiliated to a teaching hospital in Southern Nepal.

**Method:**

A 23-item questionnaire in English language with either ‘True’ or ‘False’ response as outcome was developed by reviewing findings from previous studies. Brief information was also taken to record familiarity of medical students with ECT as a treatment procedure. The study was conducted as a departmental pilot survey for quality improvement of Psychiatry Undergraduate Training. A total of 128 medical students in early clinical year enrolled in MBBS curriculum at a teaching hospital in Southern Nepal participated in the survey. The students were not exposed to any specific teaching regarding ECT while participating in the study.

**Result:**

The students were aged between 21 and 28 years with almost equal gender distribution. Among them, 89.1% had heard about ECT before whereas 15.6% knew someone who has received ECT. Although 90.6 % of students believed ECT can be lifesaving many times, a substantial number of students had misconception about ECT such as assuming it as a painful procedure (71.9%). Almost half of them believed ECT can have severe consequences like death or permanent brain damage. Around one fourth to one third believed ECT is inhumane, without scientific proof or a form of punishment for violent angry patients. Surprisingly, a significant higher percentage of male students believed that ‘ECT leads to permanent loss of memory’ (11/34 vs. 3/30, p = 0.04) and ‘ECT is given as a punishment to violent/angry patients’ (15/34 vs. 4/30, p = 0.01).

**Conclusion:**

Several misconceptions about ECT are prevalent in medical students that need to be adequately addressed during their training to develop a positive attitude and basic knowledge about the treatment.

